# *Candida* Bloodstream Infections in Critically Ill Patients: Changing Species Distribution and Mortality over a Decade in a Multidisciplinary Intensive Care Unit

**DOI:** 10.3390/pathogens15070734

**Published:** 2026-07-13

**Authors:** Maria Katsiari, Charikleia Nikolaou, Kalliopi Theodoridou, Eleftheria Palla, Athanasios Tsakris, Georgia Vrioni

**Affiliations:** 1 Intensive Care Unit, Konstantopouleio-Patission General Hospital, 3-5 Theodorou Konstantopoulou Street, N. Ionia, 14233 Athens, Greece; hariklia2009@yahoo.gr; 2 Department of Microbiology, Medical School, National and Kapodistrian University of Athens, 75 Mikras Asias Street, 11527 Athens, Greece; lmktheo@yahoo.com (K.T.); atsakris@med.uoa.gr (A.T.); 3 Department of Microbiology, Konstantopouleio-Patission General Hospital, 3-5 Theodorou Konstantopoulou Street, N. Ionia, 14233 Athens, Greece; elefthepal@gmail.com

**Keywords:** candidemia, intensive care unit, risk factors, *Candida* species, MALDI-TOF, antifungal resistance, ICU mortality

## Abstract

Introduction: Candidemia is the most significant invasive fungal disease in critically ill patients. As a shift towards non-*albicans Candida* (NAC) species has been observed in recent years, differentiation among *Candida* species is essential for appropriate therapeutic management and improved prognosis. This study evaluated trends in the epidemiology of candidemia, species-specific characteristics, treatment practices, and mortality in a multidisciplinary Greek intensive care unit (ICU). Materials and Methods: In this single-center retrospective observational study, 90 ICU patients with candidemia were evaluated. Clinical characteristics, infection-related factors, treatment practices, and outcomes were compared according to isolated *Candida* species and patient survival. Results: *C. parapsilosis* accounted for the majority of isolates (33.7%; 41.9% fluconazole-resistant), followed by *C. albicans* (31.5%) and *Candidozyma auris* (23.9%). No significant differences in co-morbidities, origin of candidemia, or treatment-related factors were identified. However, NAC bloodstream infections occurred significantly later during ICU hospitalization, were associated with lower disease severity, and were preceded by nearly five-fold higher exposure to antifungal agents before candidemia onset. Overall, the ICU mortality rate was 53.3%. No significant differences were observed among species-specific mortality rates. Neither prompt initiation of antifungal therapy nor the antifungal class administered was associated with improved survival. Conclusions: NAC species predominated among candidemia episodes in critically ill patients, with *C. parapsilosis* emerging as the most frequent isolate and exhibiting substantial fluconazole resistance. Mortality remained high and did not differ significantly according to species. Treatment timing and antifungal class were not associated with outcome, highlighting the need for continued surveillance and optimized management strategies in the ICU.

## 1. Introduction

The critically ill population encompasses patients who suffer from acute severe illness and are generally older, with comorbidities, often experiencing organ failure, and who need intensive monitoring and advanced support. These patients are vulnerable to infections, including fungal ones. *Candida* species are responsible for more than 90% of fungal bloodstream infections, and approximately one-third of these infections occur in patients admitted to the Intensive Care Unit (ICU) [1]. Indeed, candidemia is the most significant invasive fungal disease in critically ill patients, with an incidence ranging from 5.5 to 7 episodes per 1000 ICU admissions [2]. While *C. albicans* was the most prevalent etiological factor for decades, the epidemiological pattern of candidemias has altered in recent years. The shift towards *Candida* non-*albicans* species (NAC) is alarming, as many of them have intrinsic azole resistance or easily develop resistance [3,4]. Differentiating between *Candida* species is essential for the appropriate therapeutic management and improving the prognosis of candidemia. Moreover, the recently emerged *Candidozyma auris* (formerly *Candida auris*) poses a significant global threat due to its unique characteristics that include resistance to multiple antifungal agents, environmental persistence, and a strong probability of misdiagnosed strains when standard laboratory methods are used. Mortality from candidemias generally ranges from 30% to 60% and may exceed 70% among critically ill patients despite the increased use of echinocandins [5].

Nationwide studies across 28 Greek hospitals (2009–2018) placed the average incidence at 5.56 cases/100,000 inhabitants, while in the ICU setting, this rate is significantly higher. However, the incidence of *Candida* bloodstream infections increased worryingly during the COVID-19 era and *C. auris* emerged among the major causative agents [6,7]. Siopi et al. reported that candidemia incidence nearly tripled during this period, with rates reaching 94.9 episodes per 1000 COVID-19 ICU admissions [7].

We conducted a single-center retrospective observational study during a 10-year period in a Greek ICU in order to evaluate trends in epidemiology and differences among *Candida* species, along with treatment practices and mortality.

## 2. Materials and Methods

### 2.1. Setting

This retrospective observational study was conducted at the multidisciplinary nine-bed ICU of Konstantopouleio—Patission General Hospital in Athens, which is a 330-bed- tertiary care hospital that includes internal medicine, cardiology, surgical, urology, orthopedic and other wards. In this study, we studied the patients who developed candidemia between 1 January 2016 and 31 December 2025. The ICU was converted to a COVID-19 unit on 25 February 2021 during the third wave of COVID-19 in Greece and operated in this way for 15 consecutive months. Ethical review and approval for this study were in accordance with the Ethics Regulations of the hospital (Act 3096/26 January 2026). Patient consent was waived due to the observational nature of this study. The anonymity of the patients was guaranteed during the whole process of data analysis and results reporting.

### 2.2. Definitions of Cases and Collection of Clinical Data

All consecutive patients ≥18 years admitted to the ICU were considered. Candidemia was defined as the isolation of *Candida* species in at least one blood culture obtained from peripheral veins, and in the case of suspected bloodstream infection associated with a central venous catheter (CVC), additional blood cultures from the CVC were drawn.

The collection date of the first positive blood culture was set as the date of candidemia. A new episode was defined if blood cultures were positive for *Candida* species after ≥30 days from the first episode. Patients with mixed candidemia, identified as the isolation of two different *Candida* spp. from a single blood culture sample, were included.

Candidemia was defined as ICU-acquired when the patient developed candidemia 72 h or more after ICU admission. Primary candidemia comprised bloodstream infection of unknown origin in patients without an identifiable focus for the infection. A central line-associated *Candida* bloodstream infection (CLABSI) was defined as the recovery of *Candida* species from a blood culture in a patient who had a central venous catheter (CVC) at the time of infection or within 48 h before the development of infection and the candidemia must not be related to an infection from another site. Intra-abdominal source of candidemia was defined when *Candida* was isolated from intra-abdominal fluid obtained during surgery or needle aspiration, in case of peritonitis or abdominal abscess. Urinary origin of candidemia was established in cases of patients with concomitant candiduria due to the same *Candida* spp. along with urological comorbidity (obstruction or manipulation of the urinary tract).

Clinical and epidemiological data of ICU patients were reviewed, including gender, age, initial diagnosis, surgical procedures, disease severity at ICU admission as determined by Acute Physiology Chronic Health Evaluation (APACHE II) score, comorbidities (COVID-19 infection, diabetes mellitus, chronic renal failure, chronic lung disease, cardiac disease, neuropsychiatric disorder, neoplasia, immunosuppression), previous treatment with antibiotics and antifungals, outcome and length of stay (LOS) in the ICU.

At the time of candidemia, we recorded length of prior hospitalization (in a general ward or ICU), previous or current receipt of antifungals, the administration of glucocorticosteroids, and the application of central venous catheter, mechanical ventilation and renal replacement therapy.

We compared patients’ clinical characteristics and treatment-related factors among patients who survived and those who had unfavorable ICU outcome in order to determine potential predictors of mortality. Moreover, we sought possible clinical or infection-related risk factors along with differences regarding outcomes, among patients with *C. albicans* and those with NCA.

### 2.3. Identification of Isolates and Antifungal Susceptibility Testing

Yeasts isolated from blood were identified, and antifungal susceptibility testing was performed by the VITEK 2 Compact 15 automated system (Biomerieux, Marcy l’ Etoile, France) on a routine basis at the hospital clinical laboratory. Yeasts were grown on Sabouraud Dextrose agar at 35 °C and 42 °C. Additionally, CHROMagarTM *Candida* Plus agar was used, and pale cream colonies with a distinctive blue halo, suspected of being *C. auris*, were sent to the Department of Microbiology, Medical School, National and Kapodistrian University of Athens, Greece, for further analysis. Identification of isolates was confirmed by MALDI-TOF spectrometry, which is one of the most efficient diagnostic techniques for accurate identification of *C. auris*, using the Microflex LT (Bruker Daltonics, Bremen, Germany) platform. Antifungal agents used for susceptibility testing were voriconazole, fluconazole, amphotericin B, caspofungin, anidulafungin, flucytosine, micafungin. Susceptibility to antifungal agents was evaluated by the EUCAST standardized broth microdilution method and results were interpreted using the established EUCAST clinical breakpoints [https://www.eucast.org/fungi-afst/clinical-breakpoints-and-interpretation/clinical-breakpoint-table/ accessed on 10 April 2026].

### 2.4. Statistical Analysis

Continuous variables were expressed as mean ± standard deviation (SD) or median (interquartile range). Categorical variables were evaluated with the Chi-square test or Fisher’s exact test. Student’s *t*-test was used to compare normally distributed continuous variables, and the Mann–Whitney U test was used to analyze variables not normally distributed. All tests were two-tailed, and *p* < 0.05 was considered to indicate statistical significance.

## 3. Results

### 3.1. Incidence-Timeline of Cases

**Figure 1:**
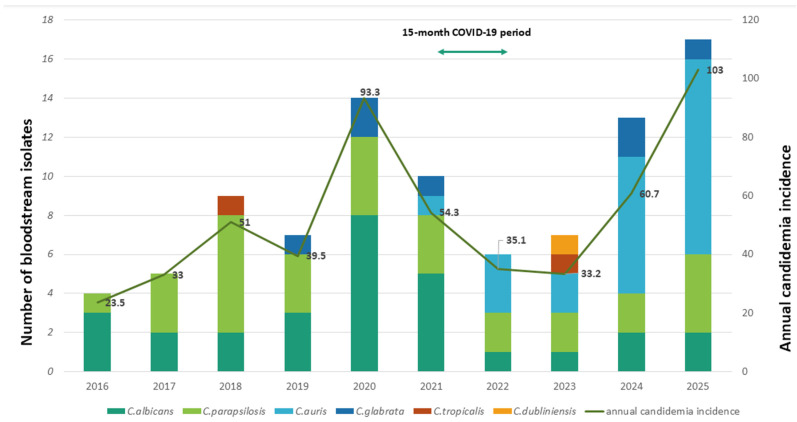
Species distribution of *Candida* bloodstream isolates during the 10-year study period (2016–2025) and annual candidemia incidence/1000 ICU admissions.

Over the 10-year study period, a total of 1737 patients were admitted to the ICU, and 90 episodes of candidemia were recorded, resulting in an overall incidence of 5.2/1000 ICU admissions/year (value for episodes/1000 ICU patient-days was 3.21). Seven candidemias occurred within 72 h after ICU admission. The median (range, IQR) number of cases emerged per year was 8 (6–11) and the highest number of cases was demonstrated in 2020 (pre-COVID-19 era; 14 cases) and in 2024–2025 (after COVID-19 era; 11 and 17 cases, respectively). No statistical difference in candidemia incidence before and after the COVID-19 era was observed (*p* = 0.614). The annual incidence rate/1000 ICU admissions and species distribution of *Candida* isolates are depicted in Figure 1.

### 3.2. Candida Species Distribution

**Figure 2:**
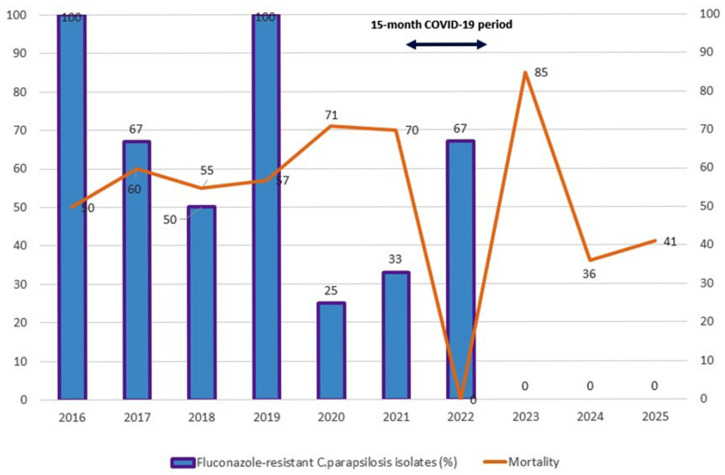
Annual ratio of fluconazole-resistant *C. parapsilosis* isolates (% of all *C. parapsilosis* isolates), and mortality (%) of the study population.

A total of 92 non-duplicate *Candida* spp. isolates were recorded; 31.5% (29/92) were *C. albicans*, and 68.5% (63/92) were NAC species. The highest *C. albicans* rate was demonstrated in 2020 (8 cases vs. 6 NAC) and decreased thereafter. Conversely, NAC species rates outweighed *C. albicans* rates, especially during 2022–2025 (Figure 1). *C. parapsilosis* accounted for the majority of isolates (33.7%; 31/92), followed by *C. albicans* (31.5%; 29/92) and *C. auris* (23.9%; 22/92). Other NAC species included *C. glabrata* (7.6%; 7/92) (former name of *Nakaseomyces glabratus*), *C. tropicalis* (2.2%; 2/92) and *C. dubliniensis* (1.1%; 1/92). Of note, *C. auris* first emerged in 2021 and predominated during 2024–2025. Regarding *C. parapsilosis* isolates, half of them (15/31) were sensitive to fluconazole (Minimum Inhibitory Concentrations; MICs ≤ 0.5–1 mg/L), whereas 3 strains exhibited MIC = 4 mg/L and 13 were fluconazole-resistant (MICs ≥ 8 mg/L). Interestingly, all resistant isolates were disseminated over the first 7 years of the study period, and during 2023–2025, no fluconazole-resistant isolates were recovered (Figure 2). All *C. auris* isolates clustered in clade I after MultiLocus Sequence Typing (MLST) of a set of four genetic loci as described previously [8], and shared almost identical susceptibilities. They were resistant to fluconazole (MICs > 32 mg/L) and susceptible to echinocandins and amphotericin B, except for one pan-echinocandin-resistant isolate that harbored an FKS1 mutation (S639F, after sequencing of the FKS1 gene), recovered from a patient on empiric therapy with anidulafungin at the end of 2025. In vitro susceptibility profile of *Candida* bloodstream isolates collected during 2016–2025 to eight antifungals is presented in Table 1.

### 3.3. Patients’ Characteristics

Each of the 90 patients suffered from one episode of candidemia. All patients had been hospitalized in a general ward and/or in the ICU before admission to our ICU [median time 4 (1–11) days]. The median age was 72 (60–82) years, and males predominated (49 patients). Concerning disease severity, the mean APACHE II score at ICU admission was 22.8 ± 8.3. The majority of patients presented with a medical reason for ICU admission (68 patients). The main comorbidities included cardiovascular disease (arterial hypertension, coronary artery disease, congestive heart failure) (59; 65.5%) and neuropsychiatric disorders (ischemic or hemorrhagic stroke, dementia, psychotic disorders) (28; 31.1%), followed by diabetes mellitus (27; 30%), and pulmonary disease (25; 27.8%). A total of 20 (22.2%) patients suffered from malignancy (solid organ or hematological).

Regarding risk factors for candidemia, all patients had at least one CVC, urinary catheter, arterial catheter, nasogastric catheter, and were on invasive mechanical ventilation. Twenty patients were on renal replacement therapy. All patients had received antibiotic treatment, whereas 21 patients (23.3%) had a recent history of antifungal therapy and 34 patients (37.8%) had a recent history of corticosteroids.

### 3.4. Infection Related Factors–Therapeutic Management

**Figure 3:**
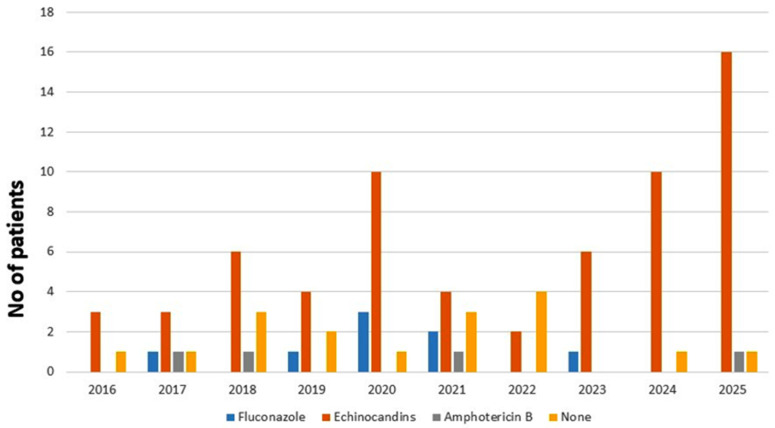
Timeline of definite treatment of candidemic patients (Amphotericin B was co-administered with echinocandins).

The majority of candidemias were classified as primary (42/90; 46.7%), followed by central-line-associated infections (37/90; 41.1%) and intra-abdominal infections (8/90; 8.9%). Seventy-three patients received antifungal treatment, and in 51 cases, treatment was initiated within 48 h after candidemia diagnosis. Echinocandins were the most frequently used antifungal agents (66/73; 90.4%), while fluconazole was administered to only seven patients (9.6%). Amphotericin B plus echinocandin was prescribed in four patients with persistent NAC candidemias (two *C. auris* and two *C. parapsilosis*) in combination with echinocandins. Concerning echinocandins, micafungin and caspofungin were the most used (27/66; 41% and 24/66; 36.4%, respectively), followed by anidulafungin (15/66; 22.7%). The timeline of definite treatment is depicted in Figure 3.

Regarding source control, CVC was promptly removed after candidemia emergence, while in cases of intra-abdominal infection, appropriate surgical procedure or drainage was performed. The urinary catheter was also replaced in cases of urinary origin of candidemia.

### 3.5. Candida Albicans vs. Non-Albicans Species

The main clinical characteristics of patients with candidemia due to *C. albicans* and NAC are depicted in Table 2. Patients with *C. albicans* candidemia presented with higher disease severity based on a significantly higher APACHE II score at ICU admission (25.9 ± 7.4 vs. 21.4 ± 8.4, *p* = 0.019). No differences concerning comorbidities and origin of candidemia were identified. However, bloodstream infection emerged significantly later during ICU hospitalization for NAC [6 (1–11) vs. 20 (3–40), *p* = 0.006]. Treatment-related factors were also similar between the two groups. Nevertheless, patients with NAC presented with almost five-fold higher receipt of antifungals before development of candidemia (6.9% vs. 31.1%, *p* = 0.023).

### 3.6. Clinical Outcomes

Median length of stay in ICU was 28 (14–64) days. All patients discharged alive from ICU, were transferred to general wards or ICUs within our or other hospitals, according to the department where each patient had been initially admitted. Median time between emergence of candidemia and ICU discharge was 14 (6–29) days and was similar in both *C. albicans* and NAC groups.

The all-cause mortality during ICU stay was 53.3% (48/90 patients), and the mortality rate per year is depicted in Figure 2. Mortality rates by species were as follows: *C. albicans* (18/29; 62%), *C. parapsilosis* (15/31; 48.4%), *C. auris* (10/22; 45.4%), *C. glabrata* (3/7; 42.8%), *C. tropicalis* (2/2; 100%), *C. dubliniensis* (0/1; 0%). No statistically significant differences were identified among species-specific mortality rates. Moreover, mortality did not significantly differ among patients with fluconazole-resistant *C. parapsilosis* candidemia (7/13; 53.8%) and fluconazole-sensitive ones (8/18; 44.4%, *p* = 0.879).

After patient allocation into two groups according to ICU outcome, sex distribution, mean age, and disease severity were similar between the groups (Table 3). Regarding comorbidities, the incidence of pulmonary disease was significantly higher in the group of deceased patients (39.6% vs. 14.3%, *p* = 0.015). Regarding associated conditions on candidemia day, the proportion of patients being on renal replacement treatment (31.25% vs. 11.9%, *p* = 0.051) was also higher in the aforementioned group. However, the difference did not reach statistical significance. Regarding treatment-related factors, neither prompt initiation of treatment nor antifungal category affected patients’ outcomes.

## 4. Discussion

We performed a retrospective observational study to analyze trends in epidemiology and mortality, along with potential risk factors, in a series of critically ill patients with candidemia during a 10-year period. *Candida* species account for more than 90% of fungal bloodstream infections among ICU patients. The multicenter EUCANDICU study estimates ICU-acquired invasive candidiasis at about 7 per 1000 admissions, while other cohorts report lower rates of candidemia (4.8/1000 ICU admissions) [9,10]. However, reported incidence rates vary significantly between countries, along with intercountry variations. Prolonged hospitalization, central venous catheterization, parenteral nutrition, broad-spectrum antibiotics, renal replacement therapy, and abdominal surgeries represent common ICU interventions that are involved in cases of invasive candidiasis [11]. In our study population, all patients shared multiple risk factors since they had at least one CVC along with urinary, arterial and nasogastric catheters, were on invasive mechanical ventilation, and had received broad-spectrum antibiotics, while 13.3% of them were on renal replacement therapy. The most frequent co-morbidities among our patients were chronic respiratory and cardiovascular diseases and diabetes mellitus, which have been linked to a high risk of early-onset ICU candidemias [12].

Incidence and distribution of *Candida* species vary within different regions, with a reported higher incidence of invasive candidiasis in Southern Europe compared with Northern or Western Europe [9,13]. *C. albicans* remains the most clinically prevalent etiological factor of invasive candidiasis in Europe [13]. However, the epidemiological pattern of candidemias has altered due to an increased rate of NAC species [5,14,15]. In our series, the candidemia rate was 5.2/1000 admissions/year, and NAC species outweighed *C. albicans*, which is consistent with previous Greek reports [16,17]. In particular, the incidence of NAC candidemias was twofold higher than *C. albicans*, with *C. parapsilosis* isolates being the most frequent ones (33.7%), followed by *C. albicans* (31.5%). Similarly, other studies found that the most frequently isolated species causing candidemias are *C. albicans* and *C. parapsilosis* [18,19].

The changes in fungal ecology are attributed to different virulence factors and resistance patterns of *Candida* species and the widespread use of antifungal agents, along with patients’ distinctive characteristics [12]. *C. albicans* remains one of the most frequent causes of early- and late-onset candidemias and emerges in blood through translocation of flora [20]. *C. parapsilosis* bloodstream infection is traditionally associated with intra-vascular catheters [21]. Moreover, surgical procedures, along with the use of total parenteral nutrition, which enhances *C. parapsilosis*’ propensity to form tenacious biofilms, have also been related to this pathogen [12,22]. *C. glabrata* affects elderly patients with underlying diseases and prior azole exposure [23]. *C. tropicalis* is more frequent in neutropenic patients with solid organ and hematological malignancies, and is also correlated with advanced age and chronic respiratory diseases [24]. *C. krusei*, rare but significant, especially affects immunocompromised patients and has an intrinsic resistance to fluconazole [25]. *C. auris*, the newest identified strain, mainly affects hospitalized and immunosuppressed patients and reveals environmental persistence, since it forms adherent biofilms and can survive on surfaces and medical equipment for more than 3 weeks [26].

In our study, the origin of candidemias along with patients’ comorbidities were similar in both *C. albicans* and NAC groups. Treatment policies and other associated conditions on candidemia day were also similar among patients of both groups. Similarly, Chow et al. found multiple common risk factors for both NAC and *C. albicans* candidemias, but they could not differentiate between these two groups based on clinical characteristics alone [27]. However, patients with *C. albicans* infection revealed higher disease severity, which is consistent with previous reports [1,28,29]. The vast majority of candidemias (92%) were classified as ICU-acquired ones. Nevertheless, NAC candidemias emerged significantly later during ICU hospitalization. An increased length of stay in the ICU might lead to a time-dependent pattern of *Candida* species distribution, which could be associated with the duration of CVC use along with administration of broad-spectrum antibiotics, antifungals and other ICU resources [30,31].

Among NAC species, *C. auris* and fluconazole-resistant *C. parapsilosis* represent major nosocomial threats due to their ability to persist in healthcare environments and to cause outbreaks, in combination with antifungal resistance patterns [32,33]. Mamali et al. reported the first nationwide study in Greece during 2009–2018, and showed that *C. parapsilosis* has emerged as a major causative agent of candidemia, accounting for 41% of isolates, especially in ICU populations, where almost half [48%] of candidemic patients were infected with *C. parapsilosis* [6]. In the present study, during 2016–2025, this trend persists since *C. parapsilosis* was the leading species. *C. parapsilosis* might be more prevalent in temperate climates compared with colder regions due to its optimal adhesion and growth at higher temperatures [1]. Nevertheless, many other factors might influence this presumed variability [34,35,36]. Worryingly, *C. parapsilosis* isolates have shown rising fluconazole resistance in a growing number of countries [3]. Mamali et al. found that almost one third (32%) of *C. parapsilosis* isolates recovered from ICU patients were fluconazole resistant, with a steadily rising isolation rate throughout 2009–2018 [6]. Since 2018, these resistant strains have been dispersed and outcompeted fluconazole-sensitive strains in Southern Europe [3]. Similarly, in our series, fluconazole resistance accounted for 42% of *C. parapsilosis* isolates. However, no resistant strains were isolated during 2023–2025, which could be attributed to local factors and practices. Given *C. parapsilosis* persistence in the environment and its ability to cause outbreaks, we could speculate that clonality of isolates might have accounted for these yearly differences. Despite its known association with fluconazole resistance, predominance of *C. parapsilosis* could be attributed to its unique capacity to persist in azole-naive environments in conjunction with high adaptability to abiotic surfaces and persistence on healthcare workers’ hands, leading to high transmissibility [3,6].

The recent evolution of *C. auris* can also be associated with the increase in global temperatures, but also with the pandemic. Indeed, a major driver of *C. auris* healthcare-associated dissemination in Greece and worldwide was COVID-19 [37,38]. Although the introduction of *C. auris* in our ICU occurred early in 2021, before the ICU’s operation as ICU- COVID-19, an outbreak was observed in the subsequent years, and finally, in 2024–2025, *C. auris* was the predominant isolate in candidemic patients [8]. *C. auris* resembles *Acinetobacter baumannii* in behavior, due to environmental persistence, disinfectant tolerance, and easy spread in healthcare facilities [11]. Therefore, the predominance of *C. auris* reinforced cleaning and disinfection procedures and adherence to hand hygiene, along with patients’ co-hosting. Meletiadis et al. reported the first pan-echinocandin-resistant isolate in Greece in 2023, recovered from a patient already receiving empiric therapy with anidulafungin [39]. Two years later, in 2025, the first pan-echinocandin-resistant strain emerged in our ICU, and until April 2026, seventeen more strains from all over Greece harbored *FKS1* mutations, mainly S639F, which were identified (data not published from the Department of Microbiology, Medical School, National and Kapodistrian University of Athens).

Changing trends in *Candida* epidemiology might account for treatment policies. The shift in the proportion of candidemia cases caused by NAC has reinforced the use of echinocandins as first-line therapy [5]. In the present study, echinocandins were the mainstay of treatment of candidemic patients. The rising percentage of fluconazole-resistant *C. parapsilosis* isolates and Clade I *C. auris* isolates that are also fluconazole-resistant might explain the extensive use of echinocandins. A recent multicenter study on antifungal prescription in Greek hospitals revealed a significantly higher echinocandins prescription in ICUs compared with other departments [40]. The recently updated guidelines of the European Confederation of Medical Mycology recommend echinocandins as first-line for candidemia, with liposomal amphotericin B and voriconazole as alternatives, and emphasize immediate catheter removal, source control, and antifungal stewardship, alongside susceptibility-guided therapy [41]. Catheter removal depicts an effective source control, either directly, in cases of catheter-related candidemias, or indirectly, in cases when the catheter is not the primary source of infection, since the secondary colonization of the catheter could perpetuate the candidemia. Interestingly, our patients with NAC candidemia presented a 4.5-fold higher percentage of prior antifungal administration compared to *C. albicans*. The longer prior ICU hospitalization of these patients could explain the administration of antifungal courses whenever a fungal infection was suspected. Moreover, patients who were exposed to azole derivatives and echinocandins were at increased risk for fungemia due to species with higher MICs to the corresponding antifungal agents [42].

In our series, 18.9% of patients did not receive antifungal treatment within the ICU setting. No specific information to justify the reasons for not treating these patients could be obtained. However, we can speculate that these patients presented a low risk of developing candidemia (nine patients) or died (eight patients) before medical staff were aware of the diagnosis. The majority of our patients (70%) received appropriate antifungal treatment within 48 h after emergence of candidemia. Nevertheless, neither prompt administration of antifungals nor antifungal category affected patients’ outcome, underscoring the importance of effective source control (e.g., CVC removal, drainage of intra-abdominal abscess, surgical debridement). Mortality in our study population accounted for 53.3%. Median time between emergence of infection and ICU discharge was 14 (6–29) and did not differ among the two outcome groups. Pulmonary disease and renal replacement therapy were more frequent among the deceased patients. These factors have been associated with candidemia risk and reflect underlying illness severity, which might hamper a favorable outcome [11,12]. Regarding species-specific mortality, although no significant differences were identified, *C. albicans* presented the highest mortality (62%), followed by *C. parapsilosis* (48.4%) and *C. auris* (45.4%). Patients with *C. albicans* displayed significantly higher disease severity based on the APACHE II score, which could explain this mortality difference. Similarly, Soriano-Martin et al. concluded that *C. parapsilosis* candidemias occurred in patients with fewer comorbidities and were associated with lower mortality than *C. albicans* cases [1]. Previous studies have demonstrated that candidemia from *C. parapsilosis* occurs in different populations than those in which candidemia from *C. albicans* occurs [18,35,43]. This difference could be attributed not only to comorbidities but also to differences in source of candidemia, given that *C. parapsilosis* and *C. auris* candidemias demonstrate a higher catheter origin, whereas *C. albicans* often originates from alterations in barrier functions and translocation of flora [12].

## 5. Limitations—Conclusions

In our 10-year period case series, we observed a shift towards non-*albicans* candidemias. *C. parapsilosis* was the most relevant pathogen (33.7%), followed by *C. albicans* (31.5%) and *C. auris* (23.9%). Non-*albicans* candidemias emerged significantly later during ICU hospitalization and were associated with lower disease severity. Despite the increased use and prompt administration of echinocandins, mortality was high (53.3%) and was similar among different species. Prolonged hospital stay due to increased survival rates, along with an increased number of transplantations and expanding indications for administration of immunosuppressive medications, has altered the at-risk population. The extensive use of indwelling catheters and other invasive procedures, along with prescription of broad-spectrum antibiotics, might further endanger ICU patients. Consequently, candidemia in ICU demands early recognition, rapid and accurate diagnostics, and antifungal stewardship at every step, in conjunction with strict implementation of infection control measures, in order to improve patients’ outcomes and diminish candidemia incidence and resistance rates of *Candida* species.

The present study has some limitations that should be considered. The observational and retrospective nature of this study brings about an intrinsic limitation. Since it is a single-center study, susceptibility patterns and management practices might have influenced our conclusions, and the results may differ according to the settings of different ICUs. Another limitation of our study is the lack of molecular characterization regarding the specific azole resistance mechanisms in the *C. auris* and *C. parapsilosis* isolates. Although mutations leading to amino acid substitutions in the *ERG11* gene are the main driver, azole resistance in both species is frequently multifactorial and involves alternative pathways, including mutations/altered expression of *TAC1B*, *ERG3*, *EFG1*, and efflux pumps [44]. Nevertheless, this study represents a real-life clinical experience that provides useful data from a contemporary overview of candidemias in critically ill patients over a 10-year period. Further prospective studies are needed to elucidate unresolved issues of *Candida* bloodstream infections, in order to improve the outcome of critically ill patients.

## Figures and Tables

**Table 1:** In vitro susceptibility profile of *Candida* bloodstream isolates collected during 2016–2025.

*Candida* spp. and Antifungal Agents	No of Tested Isolates	Clinical Breakpoints			ECOFFs *		IE ^d^
		S ^a^ No (%)	I ^b^ No (%)	R ^c^ No (%)	WT	Non-WT	
***C. albicans***							
Voriconazole	29	2 (7)	27 (93)	0			
Fluconazole	29	29 (100)	0	0			
Amphotericin B	29	29 (100)	0	0			
Flucytosine	29	-	-	-	0	2 (100)	
Caspofungin	29	28 (97)	0	1 (3)			
Micafungin	29	28 (97)	0	1 (3)			
Anidulafungin	13	13 (100)	0	0			
***C. parapsilosis***							
Voriconazole	31	21	5	5			
Fluconazole	31	15	3	13			
Amphotericin B	31	31 (100)	0	0			
Flucytosine	31	-	-	-	0	31 (100)	
Caspofungin	31	31 (100)	0	0			
Micafungin	31	31 (100)	0	0			
Anidulafungin	12	1	1	10			
***C. glabrata***							
Voriconazole	7	-	-	-	-	-	7
Fluconazole	5	0	4 (80)	1 (20)			
Amphotericin B	7	7 (100)	0	0			
Flucytosine	6	-	-	-	ND ^e^	ND ^e^	
Caspofungin	7	7 (100)	0	0			
Micafungin	7	7 (100)	0	0			
Anidulafungin	3	3 (100)	0	0			
***C. tropicalis***							
Voriconazole	2	2 (100)	0	0			
Fluconazole	2	1 (50)	0	1 (50)			
Amphotericin B	2	2 (100)	0	0			
Flucytosine	2	-	-	-	0	2 (100)	
Caspofungin	2	2 (100)	0	0			
Micafungin	2	2 (100)	0	0			
Anidulafungin	1	0	0	1 (100)			
***C. dubliniensis***							
Voriconazole	1	0	1 (100)	0			
Fluconazole	1	1	0	0			
Amphotericin B	1	1 (100)	0	0			
Flucytosine	1	-	-	-			1
Caspofungin	1	0	0	1			
Micafungin	1	0	0	1			
***C. auris***							
Voriconazole	22	0	0	22 (100)			
Fluconazole	22	0	0	22 (100)			
Amphotericin B **	22	0	22 (100)	0			
Caspofungin	22	22 (100)	0	0			
Micafungin	22	22 (100)	0	0			
Anidulafungin	22	22 (100)	0	0			

* ECOFFs: epidemiological cut-off values; ** the entire *C. auris* wild-type population is in the I category. The Susceptible category (≤0.001 mg/L) is simply to avoid misclassification of any WT strains as “S” strains; ^a^: S—Susceptible, standard dosing regimen; ^b^: I—Susceptible, increased exposure; ^c^: R—Resistant; ^d^: IE—Insufficient evidence that the organism is a good target for therapy with the agent; ^e^: ND—not determined.

**Table 2:** Clinical and infection-related characteristics of candidemic patients according to isolated *Candida* species ^a^.

Variables	All Patients (n = 90)	Patients with Non-*albicans* Candidemia (n = 61)	Patients with *C. albicans* Candidemia (n = 29)	*p*-Value
***Demographics***				
Gender (male)	49 (54.4)	35 (57.4)	14 (48.3)	0.559
Age (years) [median (IQR)]	72 (60–82)	77 (58–85)	71 (66–80)	0.583
APACHE II score (mean ± S.D.) ^b^	22.8 ± 8.3	21.4 ± 8.4	25.9 ± 7.4	**0.019**
Initial diagnosis (medical cases) ^b^	68 (75.5)	46 (75.4)	22 (75.9)	0.559
Prior LOS in hospital (days) [median (IQR)]	4 (1–11)	4 (1–10)	5 (1–14)	0.712
***Comorbidities***				
COVID-19	12 (13.3)	7 (11.5)	5 (17.2)	0.501
Cardiovascular disease	59 (65.5)	38 (62.3)	21 (72.4)	0.480
Pulmonary disease	25 (27.8)	13 (21.3)	12 (41.4)	0.083
Neuropsychiatric disease	28 (31.1)	17 (27.8)	11 (37.9)	0.472
Diabetes mellitus	27 (30)	20 (32.8)	7 (24.1)	0.555
Chronic kidney disease	13 (14.4)	9 (14.7)	4 (13.8)	1.0
Malignancy ^c^	20 (22.2)	14 (22.9)	6 (20.7)	0.976
Immunosuppression ^d^	7 (7.8)	5 (8.2)	2 (6.9)	1.0
***Associated conditions (On isolation day)***				
Abdominal surgery	22 (24.4)	15 (24.6)	7 (24.1)	0.829
ICU day [median (IQR)]	10 (2–33)	20 (3–40)	6 (1–11)	**0.006**
Corticosteroids administration (prior or currently)	34 (37.8)	20 (32.8)	14 (48.3)	0.237
Renal replacement therapy	20 (22.2)	13 (21.3)	7 (24.1)	0.976
Antifungal administration (prior or currently)	21 (23.3)	19 (31.1)	2 (6.9)	**0.023**
***Candidemia origin***				
Primary candidemia	42 (46.7)	30 (49.2)	12 (41.4)	0.640
Central line associated	37 (41.1)	25 (41)	12 (41.4)	0.847
Intra-abdominal origin	8 (8.9)	4 (6.5)	4 (13.8)	0.266
Urinary origin	3 (3.3)	2 (3.3)	1 (3.4)	1.0
***Treatment-related factors***				
Administration of antifungal treatment	73 (81.1)	50 (82)	23 (79.3)	0.998
Antifungal administration within 48 h after candidemia emergence	51 (70)	38 (76)	13 (56.5)	0.158
**First-line antifungal agent**				
Fluconazole	7 (9.6)	4 (8)	3 (13)	0.671
Echinocandin	66 (90.4)	46 (92)	20 (87)	0.671
***Outcome data***				
ICU LOS (days) [median (IQR)]	28 (14–64)	36 (13–66)	24 (14–36)	0.167
Time elapsed between 1st isolation and ICU discharge (days) [median (IQR)]	14 (6–29)	12 (6–28)	15 (7–30)	0.383
ICU mortality	48 (53.3)	30 (49.2)	18 (62)	0.358

S.D., standard deviation; APACHE, Acute Physiology and Chronic Health Evaluation; LOS, length of stay; IQR, interquartile range; ICU, intensive care unit; ^a^: data are no. (%) of patients unless otherwise stated; ^b^: at ICU admission; ^c^: solid organ malignancy, hematological malignancy; ^d^: neutropenia (neutrophil count < 1000/mm^3^), immunosuppressant medication, splenectomy.

**Table 3:** Clinical and infection-related characteristics of candidemic patients according to ICU outcome ^a^.

Variables	All Patients (n = 90)	Alive Patients (n = 42)	Deceased Patients (n = 48)	*p*-Value
***Demographics***				
Gender (male)	49 (54.4)	22 (52.4)	27 (56.25)	0.876
Age (years) [median (IQR)]	72 (60–82)	70(59–80)	77 (66–83)	0.252
APACHE II score (mean ± S.D.) ^b^	22.8 ± 8.3	21.7 ± 9.3	23.9 ± 7.3	0.211
Initial diagnosis (medical cases) ^b^	68 (75.5)	33 (78.6)	35 (72.9)	0.706
Prior LOS in hospital (days) [median (IQR)]	4 (1–11)	5(1–14)	3(1–8)	0.290
***Comorbidities***				
COVID-19	12 (13.3)	4 (9.5)	8 (16.7)	0.494
Cardiovascular disease	59 (65.5)	26 (61.9)	33 (68.75)	0.646
Pulmonary disease	25 (27.8)	6 (14.3)	19 (39.6)	**0.015**
Neuropsychiatric disease	28 (31.1)	15 (35.7)	13 (27)	0.513
Diabetes mellitus	27 (30)	14 (33.3)	13 (27)	0.678
Chronic kidney disease	13 (14.4)	4 (9.5)	9 (18.75)	0.346
Malignancy ^c^	20 (22.2)	10 (23.8)	10 (20.8)	0.932
Immunosuppression ^d^	7 (7.8)	4 (9.5)	3 (6.25)	0.701
***Associated conditions*** ***(On isolation day)***				
Abdominal surgery	22 (24.4)	9 (21.4)	13 (27)	0.706
ICU day	10 (2–33)	10(1–33)	12(3–33)	0.515
Corticosteroids administration (prior or currently)	34 (37.8)	14 (33.3)	20 (41.7)	0.551
Renal replacement therapy	20 (22.2)	5 (11.9)	15 (31.25)	0.051
Antifungal administration (prior or currently)	21 (23.3)	7 (16.7)	14 (29.2)	0.251
***Candidemia origin***				
Primary candidemia	42 (46.7)	19 (45.2)	23 (47.9)	0.966
Central line associated	37 (41.1)	20 (47.6)	17 (35.4)	0.338
Intra-abdominal origin	8 (8.9)	2 (4.8)	6 (12.5)	0.276
Urinary origin	3 (3.3)	1 (2.4)	2 (4.2)	1.0
***Pathogen-related factors***				
*Candida albicans*	29 (32.2)	11 (26.2)	18 (37.5)	0.358
Non-albicans species	61 (67.8)	31 (73.8)	30 (62.5)	0.358
***Treatment-related factors***				
Antifungal treatment	73 (81.1)	33 (78.6)	40 (83.3)	0.760
Antifungal administration within 48 h after candidemia emergence	51 (70)	22 (66.7)	29 (72.5)	0.776
**First line agent**				
Fluconazole	7 (9.6)	3 (9.1)	4 (10)	1.0
Echinocandin	66 (90.4)	30 (90.9)	36 (90)	1.0
***Outcome data***				
ICU LOS (days) [median (IQR)]	28 (14–64)	26(11–65)	33(17–60)	0.518
Time elapsed between 1st isolation and ICU discharge (days) [median (IQR)]	14 (6–29)	14 (7–30)	13 (5–27)	0.599

S.D., standard deviation; APACHE, Acute Physiology and Chronic Health Evaluation; LOS, length of stay; IQR, interquartile range; ICU, intensive care unit; ^a^: data are no. (%) of patients unless otherwise stated; ^b^: at ICU admission; ^c^: solid organ malignancy, hematological malignancy; ^d^: neutropenia (neutrophil count < 1000/mm^3^), immunosuppressant medication, splenectomy.

## Data Availability

Data available on request due to restrictions.
